# Reduced microglia Trem‐2 expression promotes LPS‐induced AD‐like pathology

**DOI:** 10.1002/alz.092583

**Published:** 2025-01-03

**Authors:** Shahnawaz A Bhat

**Affiliations:** ^1^ Aligarh Muslim University, Aligarh, UttarPradesh India

## Abstract

**Background:**

Following the genome‐wide association studies (GWAS) discovery of microglia‐specific genes, particularly Trem‐2, SHIP‐1, and CD33, significantly associated with higher Alzheimer’s disease (AD) risk, the microglia TREM2 pathway has become central for regulating amyloid load, tissue damage, and limiting its spread. These discoveries have opened up the exciting possibility of therapeutic microglia TREM2 manipulation in AD. To date, however, several elements of TREM2 signaling remain unknown, ranging from the temporal activation pattern and receptor‐ligand binding to modulation of the brain microenvironment. This study aimed to investigate the consequences of reduced microglia Trem‐2 expression on oxidative stress, inflammatory response, and cognitive impairment. We hypothesized that a decrease in Trem‐2 expression would lead to an increase in oxidative stress markers, an up‐regulation of inflammatory cytokines, and a decline in cognitive function

**Method:**

We mimicked the neuroinflammatory milieu of AD by using LPS as a neuroinflammatory tool in vitro (BV2 microglia) and in vivo (C57BL6 mice).

**Result:**

We found that LPS‐induced reduction in Trem‐2 expression resulted in significantly higher levels of oxidative stress markers, including increased production of reactive oxygen species (increased DCFDA intensity), nitrite level, and reduced GSH content, both in vitro and in vivo. Likewise, we observed a significant increase in the level of tumor necrosis factor‐alpha (TNF‐α) in both the BV2 microglia and C57BL6 mice following LPS treatment. Furthermore, the MWM cognitive test revealed a decline in spatial memory and learning abilities in mice with decreased Trem‐2 expression compared to control mice.

**Conclusion:**

These results emphasize the importance of Trem‐2 in maintaining a healthy brain environment and suggest that targeting Trem‐2 could be a potential therapeutic strategy for neurodegenerative diseases associated with microglia dysfunction.

